# Super-races are not likely to dominate a fungal population within a life time of a perennial crop plantation of cultivar mixtures: a simulation study

**DOI:** 10.1186/1472-6785-12-16

**Published:** 2012-08-03

**Authors:** Xiangming Xu

**Affiliations:** 1 State Key Laboratory of Crop Stress Biology in Arid Areas, College of Plant Protection, Northwest A&F University, Yangling 712100, China; 2 East Malling Research, New Road, West Malling, Kent ME19 6BJ, UK

**Keywords:** Mixture, Fungal diseases, Super races, Fixation, Cost of virulence, Mating system

## Abstract

**Background:**

Deployment of cultivars with different resistance in mixtures is one means to manage plant diseases and prolong the life of resistance genes. One major concern in adopting mixtures is the development of ‘super-races’ that can overcome many resistance genes present in the mixture. A stochastic simulation model was developed to study the dynamics of virulence alleles in two-cultivar mixtures of perennial crops, focusing on the effects of cost of virulence and pathogen reproduction mechanism. The simulated mechanism of virulence has characteristics of both major and minor genes.

**Results:**

Random genetic drift due to repeated population crashes during the overwintering phase led to fixation of a single fungal genotype (in terms of its virulence), often within 100 seasons. Overall, cost of virulence is most important in determining the virulence dynamics under the present model formulation. With cost of virulence incorporated, nearly all simulation runs ended up with a single fungal genotype that can infect only one of the two cultivars. In absence of cost of virulence, most of the simulation runs ended up with fungal genotypes that can infect both host cultivars but in many cases do not contain the maximum possible number of virulence alleles due to random drift. A minimum of 20% sexual reproduction between strains from different cultivars is necessary to ensure that the final fixed strains are able to infect both cultivars. Although the number of virulence alleles in the final genotype and the time to fixation are affected by simulation factors, most of the variability was among replicate simulation runs (i.e. stochastic in nature). The time to fixation is generally long relative to cropping cycles.

**Conclusions:**

A single fungal genotype will dominate a population due to the bottleneck in overwintering with cost of virulence primarily determining whether the dominant genotype can infect both cultivars. However, the dominant genotype is unlikely to accumulate all the virulence alleles due to genetic drift. The risk of emergence and spread of super-races is insufficiently great to prevent the use of cultivar mixtures of perennial crops as a means to reduce disease development provided that host resistance structure in mixtures is altered every cropping cycle.

## Background

The demand for agricultural products of high quality with little variability in harvest time has resulted in intensive monoculture of a few cultivars resistant to economically important diseases. Intensive monoculture systems are characterised by a boom-and-bust cycle: an existing variety is susceptible; a new resistant variety is developed, which introduces a boom period while the resistance lasts; the pathogen adapts itself to the resistant variety by appropriate changes to its virulence; resistance is lost and the new variety itself becomes susceptible. The boom-and-bust cycle has resulted in considerable loss of crop production and intensive use of agro-chemicals to control diseases, leading to accelerated development of fungicide resistant/insensitive pathogen strains and potential pollution of the environment. Pyramiding resistance genes is increasingly pursued to breed cultivars with durable resistance to diseases [1]. An alternative strategy to prolong the life of resistance genes is the use of cultivar mixtures, thus employing the principle of gene diversification: the planting of mixtures forces the pathogen to survive in a spatially heterogeneous host environment.

Both modelling studies [2-6] and field experiments [7-13] have shown that cultivar mixtures can significantly reduce the rate of disease increase, compared to monocultures. Disease reduction achieved by mixtures depends on the extent of autoinfection (infection resulting from inoculum produced on the same host unit), which is mainly affected by spore dispersal gradient, spatial arrangement of cultivars (genotype unit area) and number of mixture components [13-15]. Mixtures can be used to control both race-specific and non-race-specific pathogens. Race-specific pathogens are assumed to have virulence genes which match resistance factors of specific host whereas non-race-specific pathogens are assumed to have a lower degree of specificity with possible involvement of polygenic resistance [13,16].

Cultivar mixtures are a viable strategy in subsistence agriculture [17], which have been effectively exploited on a large scale in China [18]. However, mixtures are not widely used, particularly in developed countries, because of several factors. Planting mixtures may increase management cost, especially when mixture components differ in their responses to other pathogens and pests, and in their crop husbandry requirements. For some crops, planting mixtures may lead to the final product consisting of produce from several mixture components; this heterogeneity may not meet the high standard of uniformity demanded by consumers. A key biological concern of adopting mixtures is the rate of emergence of complex races (often called ‘super-races’) that can infect more mixture components than before [19,20], or the selection for pathogen strains of increased aggressiveness towards mixture components [21]. Determining the risk of emergence of super-races is particularly pertinent for perennial tree crops because frequently changing mixture components over time is not economically feasible. However, current experimental studies on cultivar mixture focus on disease reduction and do not last long enough to assess the risk of emergence of super-races over time.

The co-evolution of host and parasite of a natural system has been extensively studied, primarily through theoretical modelling, which is recently reviewed [22]; most of these modelling studies are based on the gene-for-gene (GFG) theory and focused on the outcome of this coevolution process. Modelling studies suggest that several pathogen genotypes with different virulence factors may be stably maintained [i.e. balanced polymorphism] by factors/processes that lead to uncoupling of host and parasite life cycles, such as asynchrony between host and parasite life cycles in time [23,24] and space [25]. Transient polymorphism in virulence can be promoted by both genetic and ecological factors, such as genetic drift [26] and spatial population structure [27]. The cost of virulence is one of the most important factors in maintaining polymorphisms in virulence, and hence reducing the likelihood of super-races from persisting in the population [22,28-33].

Pathogen reproduction mode (asexual and sexual) may have significant impact on host-parasite co-evolution [34,35], and has been often ignored so far in theoretical studies of host-pathogen coevolution, probably because this factor is more likely to affect the rate approaching the final outcome (equilibrium) in a natural pathosystem rather than the actual outcome of a host-pathogen co-evolution process. However, the time to the equilibrium is also important in the context of agriculture as it allows prediction of the time-scale on which a particular combination of host resistance remains effective. This is particularly true for perennial tree crops because host-resistance make-up and its spatial structure do not usually change for many years. Pathogens that undergo regular recombination, including sexual reproduction and parasexual recombination, are generally believed to pose higher risks than pathogens that undergo no or little recombination. The probability of a single isolate combining virulence alleles through sexual recombination also depends on the sexual reproduction process. For example, for pathogens without saprophytic phases it is likely more difficult for two strains with virulence genes against different resistances to mate because by definition these two strains are less likely to infect same host tissue. The relative importance of sexual and asexual spores as primary inoculum is also likely to affect the likelihood of recombination of different virulence alleles and their persistence. In addition, with a few exceptions [24,26,27] most studies ignored genetic drift, which frequently occurs for pathogens of agricultural crops in the form of repeated population crashes due to overwintering (or oversummering), crop removal (harvesting) and disease control measures.

In this study, we developed a stochastic simulation model to study the dynamics of fungal virulence in mixtures of perennial crops, focusing on the effects of cost of virulence and pathogen reproduction mode on the emergence and persistence of super-races in the population. Pathogen reproduction is characterised by the composition of sexual and asexual propagules in overwintering inocula, and the probability of the mating between strains on the same host cultivar relative to the mating between strains on different cultivars. The model extends a previous model [6] and simulates development of a generic pathogen in cultivar mixtures of perennial species.

## Results

**Figure 1:**
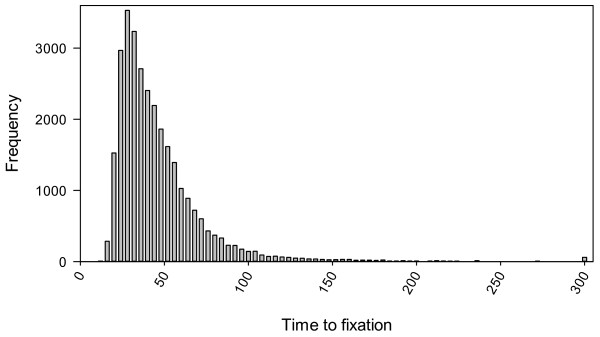
Histogram of the time to fixation (number of seasons) when all propagules are of the same genotype (with reference to fungal virulence) in the simulated fungal population.

**Figure 2:**
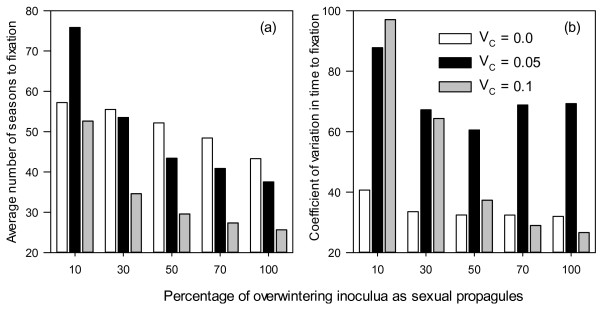
Average time (number of seasons) to fixation (a) and its coefficient of variation (b) in relation to cost of virulence and the percentage of primary inocula derived from sexual propagules.

**Figure 3:**
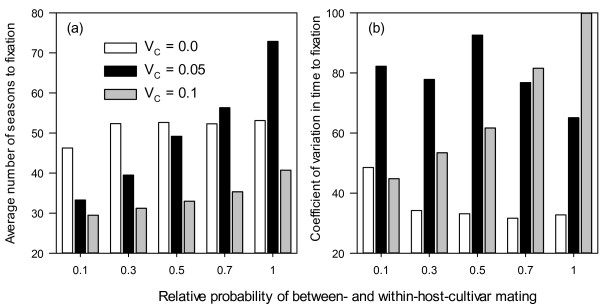
Average time (number of seasons) to fixation (a) and its coefficient of variation (b) in relation to cost of virulence and the probability of mating between diseased units from different cultivars.

Nearly 75% of simulation runs terminated within 50 seasons, i.e. there is only a single fungal genotype of virulence left in the population, and only about 4% of simulation runs terminated beyond 100 seasons (Figure 1). The longest time to fixation (T_F_) is 1012 seasons. Most of the variability (nearly 60%) in T_F_ among simulation runs cannot be explained by the simulation factors (Table 1). Of the five factors, proportion of primary inocula as sexual propagules and cost of virulence accounted for most variation (measured as deviance) in T_F_: 12.6% and 10.1%, respectively (Table 1). Increasing proportion of sexual propagules as primary inocula reduced T_F_ (Figure 2a); average T_F_ was 62, 48, 42, 39 and 35 seasons for 10%, 30%, 50%, 70% and 100% of sexual propagules in primary inocula, respectively. Increasing cost of virulence reduced T_F_, especially when sexual propagules accounted for more than 10% of primary inocula (Figure 2a). On average T_F_ was 51, 50 and 34 seasons for cost of virulence of 0.0, 0.05 and 0.1, respectively. The interaction between cost of virulence and proportion of sexual propagules accounted for 2.9% of the total variation in T_F_. The effect of between-cultivar mating was most pronounced when cost of virulence is 0.05 (Figure 3a), increasing between-cultivar mating lead to greater T_F_. Average T_F_ was 36, 41, 45, 48 and 56 seasons for between-cultivar mating of 0.1, 0.3, 0.5, 0.7 and 1.0, respectively. Variability in T_F_ among replicate runs varied greatly with simulation factors and their interactions, especially those between proportion of sexual propagules in primary inocula and cost of virulence (Figure 2b), and between cost of virulence and between-cultivar mating (Figure 3b). In general, the variability is least when there is no cost of virulence.

**Table 1:** Percentage (%) variation of the length of time to fixation, total number of virulence alleles at the fixation time, and percentage of replicate runs at the fixation with virulence against one host only explained by the main effects of and pairwise interactions between the five simulation factors (high-order interactions accounted for very little variability among simulation runs)

**Simulation variables**	**Total number of alleles (V**_**T**_**)**	**Time to Fixation (T**_**F**_**)**	**% runs with virulence against one host only (B**_**V**_**)**
Initial disease (Y_I_)^a^	0.4	1.4	0.2
Sexual primary inocula (P_C_)^b^	1.8	12.6	0.8
Between-cultivar mating (P_B_)^c^	0.8	6.4	2.6
Recombination (r)^d^	2.2	1.2	< 0.1
Cost of virulence (V_C_)^e^	36.6	10.1	77.6
P_B_ x Y_I_	< 0.1	< 0.1	0.1
P_B_ x P_C_	0.4	0.2	0.2
Y_I_ x P_C_	0.2	0.5	< 0.1
P_B_ x r	< 0.1	< 0.1	0.3
Y_I_ x r	0.2	0.2	< 0.1
P_C_ x r	0.6	0.2	0.2
P_B_ x V_C_	1.9	3.5	12.1
Y_I_ x V_C_	0.4	0.5	0.2
P_C_ x V_C_	0.9	2.9	1.6
r x V_C_	0.8	0.5	1.4
Residual	52.8	60	2.6

^a^: number of initial diseased units at the beginning of each season (96 or 192).

^b^: proportion of initial infected units (primary inocula) derived from sexual propagules (10, 30, 50, 70 and 100%).

^c^: probability of sexual propagules derived from mating between diseased units on two different hosts relative to the mating between diseased units on the same cultivar (0.1, 0.3, 0.5, 0.7 and 1).

^d^: recombination coefficients among the six virulence alleles (0.05, 0.1, 0.2 and 0.5), which were linearly located on a chromosome with three of them against the same host cultivar on the same end.

^e^: cost of virulence for each additional virulence allele against the non-host cultivar (0.0, 0.05 and 0.1).

**Figure 4:**
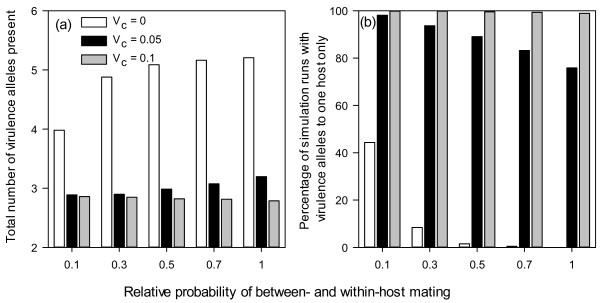
Percentage of simulation runs (a) for which at the time of fixation the final strain can only infect one cultivar, and total number of virulence alleles (b) in relation to cost of virulence and the probability of mating between diseased units from different cultivars.

Total number of virulence alleles (V_T_) at the time of fixation is mainly determined by cost of virulence, accounting for about 37% of the total variation in V_T_ (Table 1). On average there are 4.9, 3.0 and 2.8 virulence alleles in the final fixed pathogen genotype for cost of virulence of 0.0, 0.05 and 0.1, respectively. Only the interactions between proportion of sexual propagules in primary inocula and between-cultivar mating had noticeable effects on V_T_. Initial increase in between-cultivar mating led to nearly one additional virulence allele when there no cost of virulence (Figure 4a); there was an increasing difference in V_T_ between cost of virulence of 0.05 and 0.1 with increasing between-cultivar mating (Figure 4a). More than half of the variability (ca. 53%) in V_T_ among simulation runs cannot be explained by the five factors studied (Table 1).

**Figure 5:**
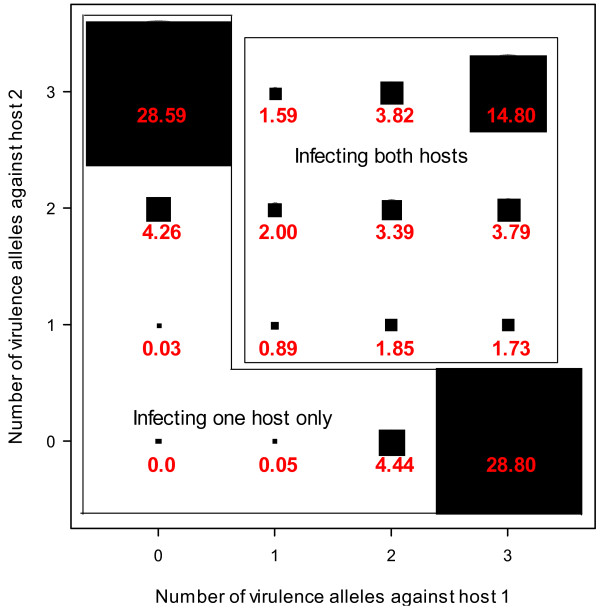
Bubble plot of the percentage of simulations with the specified composition of virulence alleles in the final fungal propagule at fixation over all simulation runs.

In contrast to T_F_ and V_T_, nearly all the variability in B_V_ was accounted for by the simulation factors, primarily by cost of virulence (78%) and its interaction with between-cultivar mating(12%) (Table 1). With larger cost of virulence (*V*_*C*_ = 0.1), nearly all simulation runs ended up with fungal propagules that can only infect one host (Figure 4b). On the other hand, at cost of virulence of 0.05 *B*_*V*_ decreased gradually from 98% to 76% when between-cultivar mating increased from 0.1 to 1.0 (Figure 4b). With no cost of virulence, *B*_*V*_ decreased sharply from 44% to 8% when between-cultivar mating increased from 0.1 to 0.3, and thereafter decreased gradually to nearly zero with increasing between-cultivar mating (Figure 4b). Overall, there were about 66% of simulation runs in which the final fixed genotype can infect only one cultivar, and only 15% of the runs in which the final fungal genotype had all six virulence alleles (Figure 5). In many simulation runs (nearly 28%), the final fungal genotype did not have the maximum possible number of virulence alleles: three in the case of infecting one cultivar only, and six in the case of infecting both cultivars (Figure 5).

## Discussion

The present study investigated fungal virulence dynamics within the context of perennial tree species in agriculture where host genetic make-up and their spatial position remained unchanged. This differs from most studies on the host-parasite co-evolution in nature [22] and annual crops. Furthermore, repeated population crashes were introduced to mimic pathogen overwintering or oversummering, which is usually not considered in GFG modelling studies. Results indicated that a single fungal genotype is likely to quickly dominate a population due to the bottleneck in overwintering with cost of virulence primarily determining whether the dominant genotype can infect both cultivars. However, the dominate genotype is unlikely to accumulate all the virulence alleles due to genetic drift. Pathogen reproduction mainly affects the time to fixation and its effects on the emergence of super-races depend on the magnitude of cost of virulence.

Nearly all simulated fungal populations became fixed within 100 seasons, probably due to genetic drift arising from repeated population crashes. Many simulated populations consisted of only two genotypes within 20 seasons, which persisted for some time before one dies out. The drift effect can also be seen in terms of the large variability in the time to fixation that cannot be explained by the simulation factors. Predicting whether the final fixed fungal genotype could overcome one or two hosts (in the case of major-gene virulence) can be made reliably as long as cost of virulence and the mechanism of sexual reproduction are known. However, the precise number of virulence alleles in the final genotype and the time to fixation are less predictable. In 28% of the simulation runs, the final fungal genotype did not possess the maximum possible number of virulence alleles due to the stochasticity in sampling overwintering inocula and generating sexual propagules. These results on the ability of infecting both cultivars and number of virulence alleles result primarily from the fact that the present model has characteristics of both major and minor genes. Any of the three virulence alleles against one cultivar behaves essentially as a major gene, as it allows the strain to infect this particular cultivar. However, subsequent addition of other virulence factors against the same cultivar behaves as a minor-gene system as it only gradually increases sporulation capacity.

Higher cost of virulence generally led to a shorter fixation time, which is probably due to strong selection against super-races leading to rapid reductions in the number of fungal genotypes in the population. On the other hand, variability among replicate runs in the fixation time was greater when there was cost of virulence, especially when the cost of virulence is intermediate. This is probably because with no cost of virulence super-races are likely to dominate quickly but at intermediate cost of virulence genetic drift plays more important role in shaping the coevolution. When cost of virulence is smaller or absent, the likelihood of emergence of super-races is also considerably affected by the mechanism of sexual reproduction. Increasing mating between fungal strains from different hosts increased the likelihood of super-races persisting in the population and becoming fixed. The present study suggests that a 20% mating chance between strains infecting different cultivars is sufficient for different virulence alleles to combine and for the resulting super-races to persist.

The probability of emergence of fungal strains with virulence against both host cultivars (i.e. the GFG theory) is primarily determined by cost of virulence and its interaction with the mechanism of sexual reproduction. With a high cost of virulence (*V*_*C*_ = 0.1), there are virtually no possibilities for fungal strains with virulence against both cultivars to persist in the population and, therefore, a super-race combining virulence alleles against both cultivars is unlikely to dominate the population. This agrees with previous modelling results that cost of virulence is likely to maintain polymorphisms in virulence. In the present study, there is no such polymorphism because of fixation arising from repeated population crashes. Cost of virulence varied considerably among pathosystems [22] and among virulence alleles of the same pathosystem [36,37]. Cost of virulence in the range of 5-10% as used in the present study is not uncommon. A cost of 20% was estimated for two virulence alleles in the wheat rust pathogen (*Puccinia striiformis* f. sp. *tritci*) [36] and about 15% for one virulence allele in *Phytophthora infestans*[38].

Pathogen reproduction mode also considerably affects evolutionary dynamics of the virulence trait. Often in population genetic modelling studies, random mating is assumed. Random mating is usually not a realistic assumption for fungi given the nature of fungal aggregation and local dispersal, often resulting in scale-dependent fungal virulence structure [39]. Sexual reproduction can happen both on attached host tissues and on dead host material and sometimes it is not certain when and where sexual reproduction has initiated. For example, although ascospores of *Venturia inaequalis* are known to be produced on fallen apple leaf debris and discharged in spring as primary inoculum [40], it is not clear whether sexual reproduction had initiated when the leaf was still attached to the tree or after leaf-fall. Mating between fungal strains that infected the same host tissue is more likely to occur, especially if sexual reproduction had initiated when the infected host tissue was still attached to the host. Since the present model did not allow multiple infections of a single host unit, the mating between strains on the same host-tissue was simulated as the mating between strains on the same cultivar. This simplification is based on the rational that strains residing on the same cultivar are more likely to have similar genetic makeup in terms of virulence than strains on different cultivars and hence more likely to infect the same host tissue than those strains from different cultivars. If the model had allowed preferential mating between strains on the same host tissue, it is even less likely that virulence alleles are combined than the present model suggested and it would probably take even longer time to fixation. The present model results may, therefore, be considered as an illustration of worst scenarios.

Inclusion of mutation and immigration will ensure generation of new fungal variability. The present study did not, however, consider mutation and immigration simply because it focuses on the likelihood of super-races dominating and persisting in the population. Thus given the fact that all virulence alleles are present in the population (but in different individual strains initially) and there is a mechanism for them to combine together, introduction of new virulence alleles in a given strain via mutation or immigration at a realistic rate should not affect the main simulation conclusions.

Time to fixation is a critical factor in determining whether the risk of emergence and subsequent spread of super-races is sufficiently great to prevent the use of cultivar mixtures. This aspect of co-evolution has so far been ignored; present study suggests that time to fixation can be greatly affected by genetic drift. The extent of population size reduction during the season depends on several factors, including amount of available alternative hosts [41,42], crop removal, and disease management practices (e.g. sanitation and fungicide use). Although the present study showed that the time to fixation is rapid in term of the evolutionary scale, it is still relatively long (in most cases more than 25 seasons) when the length of cropping cycles in agriculture is considered. For example, modern commercial apple orchards last for about 15-20 years. Therefore, it is unlikely that super-races could be fixed within one cropping cycle since the shortest fixation time occurred when there is high cost of virulence under which super-races are unlikely to persist in the population. Therefore, provided that host resistance makeup in mixtures can be altered between cropping cycles, the risk of emergence and spread of super-races should not be a major constraint for adopting mixtures.

## Conclusions

This simulation study has shown that only in the absence of cost of virulence will super-races dominate the pathogen population. Further accumulation of minor virulence genes is, however, subject more to genetic drift; consequently, in many cases the best pathogen genotype was eliminated from the fungal population by chance. Furthermore, even when super-races are eventually fixed, it took considerable length of time relative to the life of perennial agricultural crops. The risk of emergence and spread of super-races should, therefore, not be a major constraint for adopting mixtures provided that we disrupt the process of selecting super races by altering host resistance makeup between cropping cycles.

## Methods

### Description of the simulation model

The simulation model is a two-dimensional stochastic spatial contact model [43], in which spore dispersal follows a long-tailed probability distribution, and is an extension of a previous model [6]. It simulates development of a generic haploid pathogen (with a brief diploid stage during the sexual reproduction) in two-cultivar mixtures of a perennial crop species. The simulated pathogen has multiple asexual reproduction cycles but only one cycle of sexual reproduction annually. It assumes that there is no cost incurred on plant development due to disease development; this is not unrealistic for many leaf or fruit pathogens of tree crops. All model parameters and their default or range of values are given in Table 2. 

**Table 2:** Summary of model parameters, and their default or range of values that were used to generate simulations

**Model parameter**^**a**^	**Default or range of values**
Simulation grid size	240 x 240
Mixture structure	Two cultivars; alternative blocks of 20 x 20 of a single cultivar
Genotype unit area	400 plants [20 x 20]
Number of susceptible units per plant	30
Number of virulence factors against each host	3
Initial number of pathogen virulence genotypes	6 [each with one virulence factor]
Median spore dispersal distance [half-Cauchy function]	1 plant distance unit
Pathogen latent period	5 days
Pathogen infectious period	7 days
Duration of a simulated epidemics within a season	42 days
Cost of virulence for an additional virulence factor against the non-host cultivar (V_C_)	0.0, 0.05, 0.1
Sporulation rate (λ)	When V_C_ = 0, λo = 0.45, 0.50, 0.55
	When V_C_> 0, λ = λo(1 – nV_C_)
Number of initial diseased unit per fungal genotype (Y_I_)	16, 32
Proportion of sexual primary inocula (P_C_)	0.1, 0.3, 0.5, 0.7, 1.0
Probability of between-cultivar mating (P_B_) [within-cultivar mating probability is 1.0]	0.1, 0.3, 0.5, 0.7, 1.0
Recombination among virulence factors (r)	0.05, 0.1, 0.2, 0.5

This model focuses on cost of virulence and pathogen reproduction on the dynamics of virulence over many years (seasons). It did not consider pathogen infection rate, sporulation rate, spore dispersal and spatial configuration of mixture components although they have been shown previously to be important for reducing disease development [2-6] and some of them may affect host-pathogen co-evolution as well [30]. Instead, the model assumes a single value or configuration for each of these factors on the basis of previous studies [5,6]. In total, five factors (i.e., model parameters) were studied for their effects on the dynamics of virulence alleles: number of initial infected units (overwintered [primary] inocula), recombination among virulence alleles, proportion of asexual propagules in overwintered inocula, probability of mating between strains from different cultivars, and cost of virulence. All other model parameters are given a single value based on the general characteristics of a fungal airborne pathogen and previous modelling studies [5,6,44].

Disease is simulated on a rectangular grid of plants consisting of 240 rows, each with 240 plants; there are equal proportions of two cultivars that differ in their resistance. The distance between adjacent plants within a row is equal to the distance between rows: a single spatial unit. Each plant is assumed to consist of 30 units that are susceptible to the pathogen, which does not change within a season. The same number of susceptible tissue units was assumed at the beginning of each season, mimicking host regrowth. Each of the two cultivars occupies an alternative block of 20 x 20 plants in the simulation grid. To enable model results to have wide applicability, spore dispersal distances were expressed in a unit relative to the plant-to-plant distance.

#### Within-season disease development

At the start of a simulation run, an epidemic is initiated by a single infected unit (on the point of becoming infectious) on an equal number of plants of the two cultivars; these initial infected plants are located randomly within the central 204 x 204 plants of the grid (i.e., with an edge of 18 plants to reduce edge effects).

When a host unit becomes infected it enters a latent period of 5 days and becomes infectious for 7 days, producing viable spores. During the infectious period, the number of viable spores produced by each infectious unit is assumed to follow a Poisson distribution, with parameter *λ* (sporulation rate), the mean number of viable spores produced per infectious unit per day. Epidemic development is simulated at a daily step, looping through every infectious unit to simulate spore production, dispersal and infection. Spores are assumed to travel independently of one another in straight lines of random orientation with the distance varying according to a half-Cauchy distribution [45] with median dispersal distance parameter of 1.0 spatial unit. If a spore falls beyond the end of a row or beyond the extreme rows by more than 0.5 spatial units, it is ignored. Otherwise it lands on a randomly selected host unit on the nearest plant. If this host unit has already been infected previously, the spore has no effect [i.e. this model does not allow multiple infections of the same host unit]. Otherwise, if the spore has a virulence gene(s) that can overcome the host resistance (see below), this unit will be infected. At each simulation step, all infected host units (hence healthy units) were individually kept track of with all the information: the time of infection, current state (latent, infectious or non-infectious) and the time remaining in the state, pathogen genotype, and host genotype and its spatial location.

#### Host-pathogen interactions

The model assumes three independent virulence factors, one at each of three loci, against each cultivar; a recent study indicated that up to three genes may be needed for *Venturia inaequalis*(apple scab) to overcome host resistance [46]. Thus, in total there are a maximum of six virulence alleles, each at one of six loci, in a given fungal genotype against the two cultivars. At the beginning of a simulation run, there were *Y*_*I*_ initial infected units for each of the six fungal genotypes: each containing only one of the six virulence alleles. Each initial infected unit was randomly assigned to a compatible host plant, i.e. at least one of the three virulence alleles against a particular genotype is needed for successful infection (any of the three virulence alleles acts like a major gene and hence is able to overcome the host resistance). The number of virulence alleles (maximum of three) against a particular cultivar in a given fungal strain affects its sporulation rate (*λ*), simulating effects of minor genes. Without cost of virulence, *λ*_*o*_ = 0.45, 0.50 and 0.55 for strains with one, two or three virulence alleles present against the same cultivar, respectively. With cost of virulence incorporated, *λ* is proportionally reduced depending on the number of virulence alleles present against the other non-host cultivar, i.e. *λ* = *λ*_*o*_(1 – *nV*_*C*_) where *n* is the number of virulence alleles against the non-host cultivar and *V*_*C*_ is cost of virulence.

#### Generating primary inocula (between-season dynamics)

For many pathogens on perennial hosts, asexual propagules overwinter on plants (e.g. powdery mildew conidia inside dormant buds) and sexual propagules are produced on plant debris that are more or less randomly distributed in field. To simulate inoculum carry-over from the previous season, a fixed number (6*Y*_*I*_) of infected host units will be generated from the pool of infected units at the end of the previous season. A proportion (*P*_*C*_) of these inocula are allocated to sexual propagules. The remaining inocula [6*Y*_*I*_(1- *P*_*C*_)] are allocated to asexual propagules, which are randomly sampled from the pool of infected units and assigned as infected units of their original host(s) at the same spatial location(s) for the next season.

Sexual reproduction can happen both on attached host tissues and on dead host material depending on particular fungi concerned and furthermore for some pathogens it is not certain when and where sexual reproduction is initiated. Mating between strains that infected the same host tissue is more likely to occur, especially if the sexual reproduction had initiated when the infected host tissue was still attached to the host. This preferential mating is described by the probability (*P*_*B*_) of successful mating between isolates from different tissue units relative to within-unit mating. Because this model does not allow multiple infections of a single host unit, the mating between strains on the same host-tissue was simulated as the mating between strains on the same cultivar. This is because strains infecting the same cultivar are more likely to infect the same tissue units than those infecting different cultivars.

To simulate sexual reproduction, two infected units are first randomly sampled with replacement from the pool of infected units as the parental strains. If they are from the same cultivar, it proceeds to produce a sexual propagule; otherwise, it has a probability of *P*_*B*_ to mate. To generate a sexual propogule, meiosis is simulated with the simplest assumption that the six loci are linearly equally spaced on a chromosome [with an equal recombination fraction of *r*] with the three loci against the same cultivar located on the same end. A random number (0,1] was used to determine whether there is a crossover between two neighbouring loci, assuming no crossover interference. Thus for a given parental pair, five random numbers were generated to determine occurrence of crossovers between the five pairs of neighbouring loci. If the resulting propagule has no virulence alleles, the meiosis process is re-simulated until a viable propagule [i.e. with at least one of the six virulence alleles present] is generated between the two sampled strains.

The model assumes that each sexual propagule infects a unit of a randomly selected plant that is susceptible to the propagule, i.e., assuming random dispersal of sexual propagules. This process of sampling infected units for mating, generating a sexual propagule, and allocating a susceptible unit to it continues until all sexual propogules have been produced. The plants infected with primary inocula are randomly allocated to the plants in the whole simulation grid.

### Simulation experiment

Five factors were studied: number of initial infected units (primary inocula) [6*Y*_*I*_, recombination (*r*), proportion of asexual propagules in primary inocula (*P*_*C*_), probability of mating between isolates from different cultivars (*P*_*B*_) (note: within-cultivar mating is assumed to occur with a probability of 1.0), and cost of virulence (*V*_*C*_). There was a total of 96 or 192 (*Y*_*I*_ = 16, 32) initial infected units, four levels of *r* (0.05, 0.10, 0.20, 0.50), five levels of *P*_*C*_ (0.1, 0.3, 0.5, 0.7, 1.0), five levels of *P*_*B*_ (0.1, 0.3, 0.5, 0.7, 1.0), and three levels of *V*_*C*_ (0.0, 0.05 and 0.1). Thus, there were 600 combinations of five factors; for each combination, there were 50 replicate simulation runs. Within-season epidemic was simulated for 42 days; a simulated epidemic run was terminated when the pathogen population reached the fixation, i.e. fungal isolates present in the simulated population all have the same genotype in terms of their virulence trait. Random numbers were generated using the uniform random number generator of Wichmann and Hill [47].

### Analysis of simulation output

The time to fixation (T_F_, number of seasons) and the total number of virulence alleles (V_T_) in the final fungal genotype were recorded at the end of each simulation run. In addition, for each combination of the five factors, the percentage (B_V_) of simulation runs that ended up with fungal propagules that can only infect one host cultivar was calculated. Statistical analysis was conducted to assess relative contribution of each factor and their interactions to observed variation in the three variables (T_F_, V_T_ and B_V_). Ordinal regression analysis was used to assess the effects of simulation factors on V_T_. Generalised linear modelling (GLM) was used to assess the effects of the five factors on B_V_ and TF assuming that B_V_ and T_F_ follow a binomial and Poisson distribution, respectively. GLM analysis was done using Genstat^TM^[48].

## Competing interests

The author declares that he has no competing interests.
